# Body mass index is associated with the antidepressant effects of intravenous ketamine in patients with depression

**DOI:** 10.3389/fpsyt.2025.1498952

**Published:** 2025-02-18

**Authors:** Jian-Qiang Tan, Li-Mei Gu, Yan-Ling Zhou, Cheng-Yu Wang, Xiao-Feng Lan, Wei Zheng, Yu-Ping Ning

**Affiliations:** ^1^ Department of Psychiatry, The Third People's Hospital of Jiangmen, Jiangmen, China; ^2^ The Affiliated Brain Hospital, Guangzhou Medical University, Guangzhou, China; ^3^ Key Laboratory of Neurogenetics and Channelopathies of Guangdong Province and the Ministry of Education of China, Guangzhou Medical University, Guangzhou, China; ^4^ The First School of Clinical Medicine, Southern Medical University, Guangzhou, Guangdong, China

**Keywords:** body mass index, major depressive disorder, bipolar depression, intravenous ketamine, response, clinical trial

## Abstract

**Objectives:**

We aimed to explore the correlation between baseline body mass index (BMI) and the antidepressant properties of intravenous ketamine in patients with depression.

**Methods:**

We divided 135 patients diagnosed with either major depressive disorder (n=103) or bipolar depression (n=32) into lower and higher BMI groups based on their baseline BMI. Patients with a lower BMI (BMI<24 kg/m²; n=92) were included in the lower BMI group, and those with a higher BMI (BMI≥24 kg/m²; n=43) were assigned to the higher BMI group. Each participant received six ketamine infusions. Antidepressant remission was determined using a Montgomery–Åsberg Depression Rating Scale (MADRS; total score of ≤10) within 24 hours after the sixth ketamine infusion on day 13. Antidepressant response was characterized by a ≥50% alleviation in the symptoms of depression at the same time point. Changes in symptoms of depression over time were assessed using a linear mixed model.

**Results:**

The antidepressant response rate in the higher BMI group (67.4%, 95% confidence interval [CI]: 53.5%–81.4%) was higher than that in the lower BMI group (51.1%, 95% CI: 41.3%–60.9%). In addition, the remission rate was higher in the higher BMI group (39.5%, 95% CI: 25.6%–55.8%) than in the lower BMI group (31.5%, 95% CI: 21.7%–41.3%). However, these differences were not significant (all *P*>0.05). The linear mixed models with covariates indicated a significant group-by-time interaction in the MADRS scores (*F*_13, 1729_=3.0, *P<*0.001). A significant correlation was found between baseline BMI level and the change in depressive symptoms on days 13 and 26 (all *P*<0.05). However, these correlations were not significant after Bonferroni correction or controlling for baseline depressive symptoms (all *P*>0.05).

**Conclusion:**

Our exploratory, *post-hoc* analysis of an open-label, single-arm study suggests that patients with depression and a higher baseline BMI may experience greater reductions in depressive symptoms compared with those with a lower baseline BMI after receiving six ketamine infusions.

## Introduction

Depressive disorder, a complex, multifactorial disorder, is the second most common reason for years lived with disability since 2010 (1). Depression is primarily treated using pharmacotherapy; however, a considerable lag exists before the patient experiences any effect of medication (typically 2–4 weeks). Moreover, a significant proportion of patients did not achieve a response or remission until 8 weeks or later after initiating antidepressant therapy (2). Therefore, the risk of suicide and morbidity potentially increased in these patients (2), especially in the early stages of initiation of antidepressant treatment (3). Furthermore, over a third of patients with major depressive disorder (MDD) experienced treatment-resistant depression (TRD). They did not show improvement even after undergoing systematic treatment with two or more antidepressants (4). Notably, fast-acting antidepressants, such as ketamine (5–8) and esketamine (9–11), have shown encouraging antidepressant effects in patients with MDD, even TRD.

Ketamine, introduced into clinical practice in 1970, is a high-affinity, non-competitive antagonist of the N-methyl-D-aspartate receptor (NMDAR) (12). Several authors have reported the rapid antidepressant (6, 13, 14), antisuicidal (15–17), antianhedonic (18–21), and cognitive improvement (17, 22–24) effects of ketamine in patients with MDD. However, even though its antidepressant efficacy is relatively specific, 40% of patients with TRD did not achieve response efficacy after six repeated intravenous doses (13). Therefore, methods to determine the effectiveness of ketamine need to be explored.

Cognitive function, functional connectivity between brain regions, and body mass index (BMI) are recognized as predictors of the effectiveness of intravenous ketamine as an antidepressant. Better baseline visual learning (22) and lower baseline working memory (23) indicate a more robust antidepressant response after six ketamine infusions. Furthermore, changes in the functional connection between the subgenual anterior cingulate gyrus and the amygdala have been shown to predict the efficacy of antidepressant treatment (25). In addition, higher BMI and a family history of alcohol dependence have been identified as factors that may predict response to ketamine in TRD (26). Additionally, ketamine is more effective in patients with MDD and a higher BMI (BMI between 25 and 35 kg/m^2^) (27–29). Collectively, BMI appears to predict the antidepressant efficacy of intravenous ketamine. However, the correlation between BMI and the antidepressant effectiveness of intravenously administered ketamine in patients with depression has not been reported in China.

In this exploratory, *post-hoc* analysis of an open-label, single-arm study, we used the Chinese BMI classification criteria and categorized the patients into two groups based on their baseline BMI—higher BMI (BMI≥24 kg/m^2^) and lower BMI (BMI<24 kg/m^2^) groups—to determine which category of patients would exhibit better antidepressant efficacy with intravenous ketamine. Based on previous studies (27–29), we hypothesized that after six doses of intravenous ketamine, patients with depression who had a higher baseline BMI would show better antidepressant efficacy than those who had a lower baseline BMI.

## Methods

### Study design and ethics

Data from this exploratory *post hoc* study were obtained from an open-label, single-arm trial (22, 30). This study is part of an ongoing trial at the China Clinical Trial Registry (registration number: ChiCTR-OOC-17012239), officially initiated at the Affiliated Brain Hospital, Guangzhou Medical University, in November 2016. The study protocol was approved by the Ethics Committee of the Affiliated Brain Hospital, Guangzhou Medical University (Ethics Application No. 2016030) and was in accordance with the principles of the Declaration of Helsinki. Written informed consent was obtained from all participating patients.

### Patient selection

According to the Diagnostic and Statistical Manual of Mental Disorders, Fifth Edition (DSM-5), individuals aged 18–65 with different BMIs diagnosed with MDD or bipolar depression (BD) and without psychotic symptoms were eligible to participate in this study. Additionally, patients should obtain a baseline score of ≥17 on the 17-item Hamilton Depression Rating Scale (HAMD-17) to confirm their experience with TRD and/or suicidal ideation (31). TRD was defined as being unresponsive to systemic treatment with two or more antidepressants in adequate doses and regimens (32). A score of ≥2 on part 1 of the Beck Scale of Suicidal Ideation (SSI) confirms suicidal ideation (33). The complete inclusion and exclusion criteria used in this study are available in previously published reports (22, 30).

### Ketamine infusions

The study protocol was derived from the previous studies (5, 34). Briefly, 135 patients who met the inclusion criteria received six doses of ketamine (0.5 mg/kg) intravenously three times per week, administered via an intravenous pump over two weeks. The infusion was administered over 40 minutes, and a trained psychiatrist monitored the vital signs of the patient every 10 minutes. Subsequently, the patient remained in the treatment room for at least 30 minutes for observation. Patients were allowed to continue taking their previously prescribed psychiatric medications during this study.

### Clinical assessment

A standardized data collection form was used to collect demographic and clinical data after patient enrollment. The Montgomery– Åsberg Depression Rating Scale (MADRS) was used to assess depressive symptom severity (35). MADRS evaluations were conducted at baseline (day 0), 4 and 24 hours after each intravenous ketamine injection, and two weeks post-infusion (day 26). The inter-evaluator correlation coefficient was greater than 0.9. The primary outcome was the association between baseline BMI level and the antidepressant effects of intravenous ketamine in patients with depression. The secondary outcomes included: 1) the antidepressant response rate, defined as a ≥50% reduction in the total MADRS score from day 0 to day 13; 2) the antidepressant remission rate, defined as a total MADRS score of ≤10 at 24 hours after the sixth ketamine infusion (day 13) (36); and 3) the changes in MADRS scores.

### BMI measures

BMI, a simple anthropometric measure, is commonly used to assess the presence and severity of excess body fat in adults, and it is defined as the weight in kilograms divided by the square of the height in meters (37). The height and weight of participants were measured after enrollment. They were asked to stand on a weighing scale (instrument model: RGZ-120-RT). The Chinese Working Group on Obesity (WGOC) defines BMIs of ≥24 kg/m² as high BMI (38). Asians tend to have a higher percentage of body fat at lower BMIs than Europeans (39, 40). Moreover, a BMI of 24 kg/m^2^ has been widely used as a threshold in Asians in previous studies (41–43). Therefore, we categorized patients with BMIs of <24 kg/m^2^ and ≥24 kg/m^2^ into lower and higher BMI groups, respectively.

### Statistical analysis

A two-sided *P* value of 0.05 was considered statistically significant and determined using IBM Statistical Product and Service Solutions (SPSS) version 25.0. In descriptive analyses, continuous variables, including age, education, duration of illness, age of onset, baseline HAMD-17 scores, baseline MADRS scores, and baseline BMI, were presented as mean, range, and standard deviation (SD). Comparisons of these variables were performed using the Student's t-test or the Mann–Whitney U-test. Categorical variables, such as gender, marital status, work status, living alone status, previous hospitalization, family history of psychosis, antidepressant responders and remitters, were expressed as frequency, percentages (%), and the 95% confidence interval (CI) of percentages. They were analyzed using the chi-square test or Fisher's exact test. Changes in MADRS scores from day 0 to day 26 were assessed using a linear mixed model. The baseline of MADRS scores and the variates (excluding baseline BMI) that showed significant differences between the two groups in demographic or clinical characteristics were included as covariates in the linear mixed model. BMI subgroup, time point, the interaction of BMI subgroup and time point, and covariates entered the linear mixed model as fixed effects. The time point entered the model as a random effect. Bonferroni corrections were applied to account for multiple tests. The associations between changes in depressive symptoms and baseline BMI levels were examined using Spearman's correlation analysis.

## Results

**Table 1** presents the demographic and clinical characteristics of the patients. We included 135 patients diagnosed with MDD (n=103) or BD (n=32) with an average age of 34.8 years in the final analysis. Patients with a higher BMI (n=43) were older (*P*=0.033), predominantly male (*P*=0.007), less likely to be employed (*P*=0.035), and more likely to report a family history of mental illness (*P*=0.028) compared with those with a lower BMI (n=92).

**Table 1:** Demographic, clinical characteristics, and the percentage of antidepressive response and remission in depressed patients with lower versus higher BMI.

Variables	Lower BMI (n=92)		Higher BMI (n=43)		Statistics		
	N	% (95% CI)	N	% (95% CI)	χ^2^	df	*P*
Male	39	42.4 (32.6-53.3)	29	67.4 (53.5-81.4)	7.4	1	**0.007**
Married	45	48.9 (39.1-59.8)	26	60.5 (46.5-74.4)	1.6	1	0.210
Employed	41	44.6 (34.8-54.3)	11	25.6 (14.0-39.5)	4.5	1	**0.035**
Living alone	6	6.5 (2.2-12.0)	5	11.6 (2.3-20.9)	0.5	1	0.501
No history of psychiatric hospitalization	66	71.7 (62.0-81.5)	27	62.8 (48.8-76.7)	1.1	1	0.295
Having a family history of psychiatric disorders	29	31.5 (21.7-41.3)	22	51.2 (37.2-67.4)	4.8	1	**0.028**
Comorbidities	12	13.0 (6.5-20.7)	11	25.6 (11.6-37.2)	3.3	1	0.071
TRD	15	16.3 (8.7-23.9)	8	18.6 (7.0-30.2)	0.1	1	0.740
Antidepressant responders	47	51.1 (41.3-60.9)	29	67.4 (53.5-81.4)	3.2	1	0.074
Antidepressant remitters	29	31.5 (21.7-41.3)	17	39.5 (25.6-55.8)	0.8	1	0.360
	Mean (Range)	SD	Mean (Range)	SD	T/Z	df	*P*
Age (years)	33.5 (18-65)	11.6	37.6 (20-63)	11.7	-2.1	---^a^	**0.033**
Education (years)	12.6 (3-16)	3.1	11.4 (5-17)	3.5	-1.8	---^a^	0.072
Age of onset (years)	25.4 (10-57)	11.1	27.9 (11-61)	12.3	-1.1	---^a^	0.256
Duration of illness (months)	93.1 (1-408)	84.1	121.0 (1-372)	105.0	-1.3	---^a^	0.182
Baseline HAMD-17 scores	23.5 (17-38)	5.1	24.4 (17-38)	5.1	-1.1	---^a^	0.257
Baseline MADRS scores	31.9 (13-50)	7.6	33.9 (17-56)	8.7	-1.4	133	0.175
Baseline BMI	20.6 (15.2-23.8)	2.0	26.7 (24.0-33.2)	2.2	9.3	---^a^	**<0.001**

a Mann–Whitney U test.

Bolded values are *P*<0.05.

BMI, body mass index; df, degree of freedom; HAMD-17, 17-item Hamilton Rating Scale for Depression; MADRS, Montgomery-Åsberg Depression Rating Scale; SD, standard deviation; TRD, treatment-resistant depression; 95% CI, 95% Confidence Interval.

**Figure 1:**
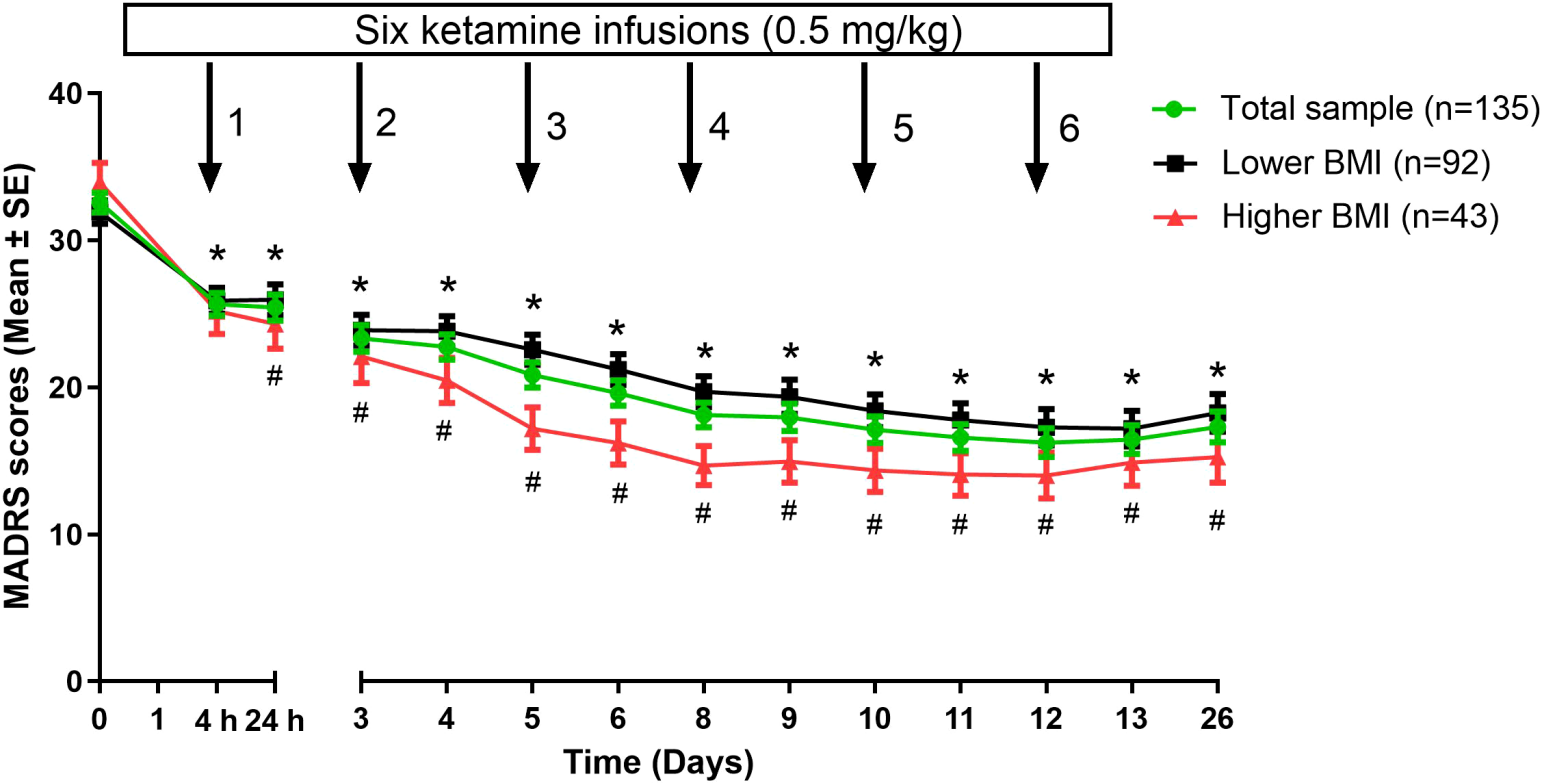
Changes in depressive symptoms of depressed patients with lower versus higher BMI following multiple ketamine infusions. *Significant differences were found when comparing the baseline at the indicated times in either lower BMI group or higher BMI group (*P*<0.05). ^#^Significant differences were found between depressed patients with lower and higher BMI at the indicated times (*P*<0.05). BMI, body mass index; MADRS, Montgomery-Åsberg Depression Rating Scale; SE, standard error.

The antidepressant response rate was higher in the higher BMI group (67.4%, 95% CI: 53.5%–81.4%) than in the lower BMI group (51.1%, 95% CI: 41.3%–60.9%). In addition, the remission rate was higher in the higher BMI group (39.5%, 95% CI: 25.6%–55.8%) than in the lower BMI group (31.5%, 95% CI: 21.7%–41.3%). However, these differences were not significant (all *P*>0.05; **Table 1**). When depressive symptom changes over time were analyzed using a linear mixed model with covariates, significant differences were observed in time effect (*F*_13, 1729_=77.8, *P*<0.001; **Table 2**), group effect (*F*_1, 128_=12.8, *P*<0.001; **Table 2**), and group-by-time interaction (*F*_13, 1729_=3.0, *P*<0.001; **Table 2**). Both groups showed that depressive symptoms significantly reduced over time from the first to sixth injections and on day 26 compared to baseline (all *P*<0.05; **Figure 1**). Furthermore, patients with a higher baseline BMI showed a more pronounced reduction in depressive symptoms than those with a lower baseline BMI from 24 hours after the first injection through day 26 (all *P*<0.05; **Figure 1**).

**Table 2:** Comparison of MADRS scores between the lower and higher BMI groups using linear mixed model analysis.

Variables	Time effect		Group effect		Time × group interaction	
	*F_1_*_3,1729_	*P*	*F*_1,128_	*P*	*F_1_*_3,1729_	*P*
MADRS	77.8	**<0.001**	12.8	**<0.001**	3.0	**<0.001**

Bolded values are *P*<0.05.

BMI, body mass index; MADRS, Montgomery-Åsberg Depression Rating Scale.

Baseline BMI levels were not significantly associated with depressive symptoms on days 13 or 26 (all *P*>0.05; **Table 3**). Notably, a significant correlation was found between baseline BMI levels and the change in depressive symptoms on day 13 and 26 (all *P*<0.05; **Table 3**). However, these correlations were not significant after Bonferroni correction or controlling for baseline depressive symptoms (all *P*>0.05).

**Table 3:** Correlation analysis between baseline BMI levels and depressive symptoms in depressed patients following ketamine treatments.

Variables		Depressive symptoms^a^			Change in depressive symptoms^a^	
		At Baseline	At Day 13	At Day 26	At Day 13	At Day 26
Baseline BMI levels	r	0.111	-0.046	-0.110	-0.170	-0.203
	*P*	0.202	0.595	0.203	0.049^b^	0.018^b^

a Depressive symptoms were assessed by the MADRS.

b The significance was not maintained after Bonferroni correction or controlling for baseline depressive symptoms.

Bolded values are *P*<0.05.

BMI, body mass index; MADRS, Montgomery-Åsberg Depression Rating Scale; r, Spearman coefficient of correlation.

## Discussion

To our knowledge, this exploratory, *post-hoc* analysis of an open-label, single-arm study reported the correlation between baseline BMI and the antidepressant effects of a subanesthetic dose of ketamine (0.5 mg/kg for 40 min) in patients with depression in China. The primary findings of this study suggest that ketamine quickly alleviates depressive symptoms in patients with depression, showing comparable antidepressant responses and remission rates across both BMI groups. Additionally, the higher BMI group exhibited lower levels of depressive symptoms compared with the lower BMI group on days 13 and 26. The correlation between baseline BMI and changes in depressive symptoms on days 13 and 26 was significant. However, these correlations were not significant after Bonferroni correction or controlling for baseline depressive symptoms.

Lipsitz et al. observed comparable antidepressant response and remission rates across four BMI categories (normal: 18.0–24.9 kg/m^2^, overweight: 25–29.9 kg/m^2^, obese I: 30–34.9 kg/m^2^, and obese II: ≥35.0 kg/m^2^) (44). Similar to the findings of Lipsitz et al. (44), our results indicated similar antidepressant effects with subanesthetic doses of ketamine across both BMI groups. While BMI may not be a reliable predictor for the antidepressant effects of conventional monoamine-based antidepressants such as sertraline, Lipsitz et al. reported an association between BMI and the antidepressant effects of subanesthetic doses of ketamine (44). Given that ketamine primarily targets the glutamate system instead of monoaminergic system targeted by conventional antidepressants, our findings highlight a unique pattern. We observed that patients with depression and higher BMI levels experienced a more significant reduction in depressive symptoms after ketamine treatment than those with lower BMI levels.

However, studies examining the association between BMI and the antidepressant effects of ketamine have yielded inconsistent findings (26, 45–47). For instance, Niciu et al. (46) reported that patients with TRD and higher baseline BMI experienced markedly greater improvement in depressive symptoms at both 230 minutes and day 1 after a subanesthetic intravenous ketamine infusion (0.5 mg/kg) than those with lower BMI levels. In contrast, Machado-Vieira et al. (45) reported an inverse relationship, suggesting that lower adiponectin levels at baseline could predict the antidepressant effects of ketamine in patients with MDD or BD. Lipsitz et al. (44) found that baseline BMI levels were not predictive of the response to four ketamine infusions. Dale et al. suggested that metabolic syndrome instead of BMI may predict antidepressive response of ketamine (47). Considering these variable findings, our findings indicate a positive correlation between BMI in patients with depression and the anti-depressive effects of ketamine. The inconsistent results in different studies may be partly due to differences in the ketamine administration protocol, sample size, outcome measures, or duration of follow-up in these trials (44–47). For example, repeated ketamine infusions were administered by Dale et al. (47) and Lipsitz et al. (44), whereas a single ketamine infusion was administered by Niciu et al. (46) and Machado-Vieira et al. (45). Further research is needed to validate and expand upon our preliminary findings.

Patients with depression are prone to comorbid obesity, and these conditions can exacerbate each other (48, 49). Leptin, an adipokine produced by adipose tissue, has antidepressant effects (50). Notably, patients with higher BMI, especially those who are obese or overweight, are more likely to experience leptin resistance (51). The antidepressant effects of ketamine may bypass this functional deficit caused by leptin resistance through its action on the glutamatergic system, potentially explaining why ketamine may be more effective in individuals with higher BMI (52). Additionally, several studies have explored the relationship between inflammatory factors and changes in depressive symptoms after intravenous ketamine treatment, but with inconsistent findings (53–56). For instance, a reduction in tumor necrosis factor-alpha (TNF-α) levels from baseline to 40 minutes after the administration of subanesthetic doses of intravenous ketamine (0.5 mg/kg) was positively associated with decreased MADRS scores over time in patients with TRD (53). Similarly, when patients with TRD received six infusions of ketamine, the reduction in interleukin-6 (IL-6) levels was positively associated with a reduction in depressive symptoms (54). Park et al. observed increased IL-6 levels at 230 minutes after ketamine infusion in patients with TRD; however, the change in IL-6 levels at 230 minutes after ketamine infusion was not related to the change in MADRS score at 230 minutes (55). Similarly, Kruse and colleagues found no significant relationships between the antidepressant effects of ketamine and IL-6 and TNF-α levels in patients with depression after receiving an infusion of ketamine (0.5 mg/kg infused intravenously over 40 minutes) (56). However, inflammatory factors (such as TNF-α and IL-6) were not analyzed in this study.

Our study had several limitations. First, this study was conducted with a relatively small sample size (n=135) at a single center, limiting the generalization of our findings to other psychiatric facilities. Second, the absence of a placebo control in this open-label, single-arm trial could lead to biases, such as the placebo effect and observer expectations, which might impact the reliability of the findings. Third, the absence of measurements for biomarkers, such as inflammatory cytokines or adipokines, limits the understanding of the biological mechanisms underlying the observed effects in this study. In psychiatric diagnostics, structured interviews are essential for ensuring reliable and valid assessments. However, the prominent tools in this domain, such as the Mini-International Neuropsychiatric Interview (MINI), were not used in this study. Fourth, we applied a BMI cutoff of 24 kg/m² according to Chinese classification standards. However, it is uncertain whether the ketamine response differs when using this BMI cutoff compared to other cutoffs, such as the internationally common 25 kg/m². Finally, the participants continued taking the psychotropic medications prescribed by their psychiatrists before the beginning of the trial. This ongoing medication regimen could potentially influence the antidepressant effects observed with ketamine treatment.

## Conclusion

Our exploratory, *post-hoc* analysis of an open-label, single-arm study suggests that patients with depression and a higher baseline BMI may experience greater reductions in depressive symptoms compared with those with a lower baseline BMI after receiving six ketamine infusions. However, these findings should be validated through a randomized controlled trial with a more extensive sample size.

## Data Availability

The data used and analyzed during the current study are available from the corresponding author upon reasonable request.

## References

[1] Lu J Xu X Huang Y Li T Ma C Xu G. Prevalence of depressive disorders and treatment in China: a cross-sectional epidemiological study. Lancet Psychiatry. (2021) 8:981–90. doi: 10.1016/S2215-0366(21)00251-0 34559991

[2] Trivedi MH Rush AJ Wisniewski SR Nierenberg AA Warden D Ritz L. Evaluation of outcomes with citalopram for depression using measurement-based care in star*d: implications for clinical practice. Am J Psychiatry. (2006) 163:28–40. doi: 10.1176/appi.ajp.163.1.28 16390886

[3] Jick H Kaye JA Jick SS. Antidepressants and the risk of suicidal behaviors. Jama. (2004) 292:338–43. doi: 10.1001/jama.292.3.338 15265848

[4] Ruberto VL Jha MK Murrough JW. Pharmacological treatments for patients with treatment-resistant depression. Pharmaceuticals. (2020) 13:116. doi: 10.3390/ph13060116 32512768 PMC7345023

[5] aan het Rot M Collins KA Murrough JW Perez AM Reich DL Charney DS. Safety and efficacy of repeated-dose intravenous ketamine for treatment-resistant depression. Biol Psychiatry. (2010) 67:139–45. doi: 10.1016/j.biopsych.2009.08.038 19897179

[6] Dwyer JB Landeros-Weisenberger A Johnson JA Londono Tobon A Flores JM Nasir M. Efficacy of intravenous ketamine in adolescent treatment-resistant depression: a randomized midazolam-controlled trial. Am J Psychiatry. (2021) 178:352–62. doi: 10.1176/appi.ajp.2020.20010018 33653121

[7] Zheng W Zhou YL Liu WJ Wang CY Zhan YN Lan XF. A preliminary study of adjunctive ketamine for treatment-resistant bipolar depression. J Affect Disord. (2020) 275:38–43. doi: 10.1016/j.jad.2020.06.020 32658821

[8] Zheng W Zhou YL Wang CY Lan XF Zhang B Zhou SM. Plasma bdnf concentrations and the antidepressant effects of six ketamine infusions in unipolar and bipolar depression. PeerJ. (2021) 9:e10989. doi: 10.7717/peerj.10989 33850645 PMC8015784

[9] Jawad MY Di Vincenzo JD Ceban F Jaberi S Lui LMW Gillissie ES. The efficacy and safety of adjunctive intranasal esketamine treatment in major depressive disorder: a systematic review and meta-analysis. Expert Opin Drug safe. (2022) 21:841–52. doi: 10.1080/14740338.2022.2058488 35387538

[10] McIntyre RS Rosenblat JD Nemeroff CB Sanacora G Murrough JW Berk M. Synthesizing the evidence for ketamine and esketamine in treatment-resistant depression: an international expert opinion on the available evidence and implementation. Am J Psychiatry. (2021) 178:383–99. doi: 10.1176/appi.ajp.2020.20081251 PMC9635017 33726522

[11] Zheng W Cai DB Xiang YQ Zheng W Jiang WL Sim K. Adjunctive intranasal esketamine for major depressive disorder: a systematic review of randomized double-blind controlled-placebo studies. J Affect Disord. (2020) 265:63–70. doi: 10.1016/j.jad.2020.01.002 31957693

[12] Nowacka A Borczyk M. Ketamine applications beyond anesthesia - a literature review. Eur J Pharmacol. (2019) 860:172547. doi: 10.1016/j.ejphar.2019.172547 31348905

[13] Phillips JL Norris S Talbot J Birmingham M Hatchard T Ortiz A. Single, repeated, and maintenance ketamine infusions for treatment-resistant depression: a randomized controlled trial. Am J Psychiatry. (2019) 176:401–9. doi: 10.1176/appi.ajp.2018.18070834 30922101

[14] Zheng W Zhou Y-L Liu W-J Wang C-Y Zhan Y-N Li H-Q. Investigation of medical effect of multiple ketamine infusions on patients with major depressive disorder. J psychopharmacol. (2019) 33:494–501. doi: 10.1177/0269881119827811 30789302

[15] Zhan Y Zhang B Zhou Y Zheng W Liu W Wang C. A preliminary study of anti-suicidal efficacy of repeated ketamine infusions in depression with suicidal ideation. J Affect Disord. (2019) 251:205–12. doi: 10.1016/j.jad.2019.03.071 30927581

[16] Abbar M Demattei C El-Hage W Llorca PM Samalin L Demaricourt P. Ketamine for the acute treatment of severe suicidal ideation: double blind, randomised placebo controlled trial. Bmj. (2022) 376:e067194. doi: 10.1136/bmj-2021-067194 35110300 PMC8808464

[17] Chen X Wang M Hu Y Zhan Y Zhou Y Zheng W. Working memory associated with anti-suicidal ideation effect of repeated-dose intravenous ketamine in depressed patients. Eur Arch Psychiatry Clin Neurosci. (2021) 271:431–8. doi: 10.1007/s00406-020-01221-z 33386430

[18] Delfino RS Del-Porto JA Surjan J Magalhaes E Sant LCD Lucchese AC. Comparative effectiveness of esketamine in the treatment of anhedonia in bipolar and unipolar depression. J Affect Disord. (2021) 278:515–8. doi: 10.1016/j.jad.2020.09.056 33017679

[19] Zheng W Gu LM Sun CH Zhou YL Wang CY Lan XF. Comparative effectiveness of repeated ketamine infusions in treating anhedonia in bipolar and unipolar depression. J Affect Disord. (2022) 300:109–13. doi: 10.1016/j.jad.2021.12.105 34965393

[20] Zheng W Gu L Zhou Y Wang C Lan X Zhang B. Baseline plasma bdnf levels are associated with antianhedonic effects of repeated-dose intravenous ketamine in major depressive disorder. Curr neuropharmacol. (2022) 21:1013–21. doi: 10.2174/1570159x20666220927085706 PMC10227912 36173064

[21] Zheng W Yang XH Gu LM Tan JQ Zhou YL Wang CY. Antianhedonic effects of serial intravenous subanaesthetic ketamine in anxious versus nonanxious depression. J Affect Disord. (2022) 313:72–6. doi: 10.1016/j.jad.2022.06.081 35772627

[22] Zhou Y Zheng W Liu W Wang C Zhan Y Li H. Neurocognitive effects of six ketamine infusions and the association with antidepressant response in patients with unipolar and bipolar depression. J psychopharmacol. (2018) 32:1118–26. doi: 10.1177/0269881118798614 30260273

[23] Zheng W Zhou YL Wang CY Lan XF Zhang B Yang MZ. Neurocognitive effects of six ketamine infusions and the association with antidepressant effects in treatment-resistant bipolar depression: a preliminary study. PeerJ. (2020) 8:e10208. doi: 10.7717/peerj.10208 33194410 PMC7646297

[24] Basso L Bonke L Aust S Gartner M Heuser-Collier I Otte C. Antidepressant and neurocognitive effects of serial ketamine administration versus ect in depressed patients. J Psychiatr Res. (2020) 123:1–8. doi: 10.1016/j.jpsychires.2020.01.002 31981856

[25] Nakamura T Tomita M Horikawa N Ishibashi M Uematsu K Hiraki T. Functional connectivity between the amygdala and subgenual cingulate gyrus predicts the antidepressant effects of ketamine in patients with treatment-resistant depression. Neuropsychopharmacol Rep. (2021) 41:168–78. doi: 10.1002/npr2.12165 PMC8340826 33615749

[26] Rong C Park C Rosenblat JD Subramaniapillai M Zuckerman H Fus D. Predictors of response to ketamine in treatment resistant major depressive disorder and bipolar disorder. Int J Environ Res Public Health. (2018) 15:771. doi: 10.3390/ijerph15040771 29673146 PMC5923813

[27] Chen MH Wu HJ Li CT Lin WC Bai YM Tsai SJ. Using classification and regression tree modelling to investigate treatment response to a single low-dose ketamine infusion: *post hoc* pooled analyses of randomized placebo-controlled and open-label trials. J Affect Disord. (2021) 281:865–71. doi: 10.1016/j.jad.2020.11.045 33239245

[28] Freeman MP Hock RS Papakostas GI Judge H Cusin C Mathew SJ. Body mass index as a moderator of treatment response to ketamine for major depressive disorder. J Clin psychopharmacol. (2020) 40:287–92. doi: 10.1097/jcp.0000000000001209 PMC7185034 32332464

[29] Singh B Bobo WV Rasmussen KG Stoppel CJ Rico JA Schak KM. The association between body mass index and remission rates in patients with treatment-resistant depression who received intravenous ketamine. J Clin Psychiatry. (2019) 80:19l12852. doi: 10.4088/JCP.19l12852 31721482

[30] Zheng W Zhou YL Liu WJ Wang CY Zhan YN Li HQ. Rapid and longer-term antidepressant effects of repeated-dose intravenous ketamine for patients with unipolar and bipolar depression. J Psychiatr Res. (2018) 106:61–8. doi: 10.1016/j.jpsychires.2018.09.013 30278319

[31] Hamilton M. A rating scale for depression. J Neurol Neurosurg Psychiatry. (1960) 23:56–62. doi: 10.1136/jnnp.23.1.56 14399272 PMC495331

[32] Diamond PR Farmery AD Atkinson S Haldar J Williams N Cowen PJ. Ketamine infusions for treatment resistant depression: a series of 28 patients treated weekly or twice weekly in an ect clinic. J psychopharmacol. (2014) 28:536–44. doi: 10.1177/0269881114527361 24699062

[33] Beck AT Kovacs M Weissman A. Assessment of suicidal intention: the scale for suicide ideation. J consult Clin Psychol. (1979) 47:343–52. doi: 10.1037//0022-006x.47.2.343 469082

[34] Shiroma PR Johns B Kuskowski M Wels J Thuras P Albott CS. Augmentation of response and remission to serial intravenous subanesthetic ketamine in treatment resistant depression. J Affect Disord. (2014) 155:123–9. doi: 10.1016/j.jad.2013.10.036 24268616

[35] Montgomery SA Asberg M. A new depression scale designed to be sensitive to change. Br J psych: J Ment sci. (1979) 134:382–9. doi: 10.1192/bjp.134.4.382 444788

[36] Zimmerman M Posternak MA Chelminski I. Derivation of a definition of remission on the montgomery-asberg depression rating scale corresponding to the definition of remission on the Hamilton rating scale for depression. J Psychiatr Res. (2004) 38:577–82. doi: 10.1016/j.jpsychires.2004.03.007 15458853

[37] Sweatt K Garvey WT Martins C. Strengths and limitations of bmi in the diagnosis of obesity: what is the path forward? Curr Obes Rep. (2024) 13:584–95. doi: 10.1007/s13679-024-00580-1 PMC11306271 38958869

[38] Ji CY Chen TJ. Empirical changes in the prevalence of overweight and obesity among chinese students from 1985 to 2010 and corresponding preventive strategies. Biomed Environ sci: BES. (2013) 26:1–12. doi: 10.3967/0895-3988.2013.01.001 23294610

[39] Zeng Q He Y Dong S Zhao X Chen Z Song Z. Optimal cut-off values of bmi, waist circumference and waist:height ratio for defining obesity in chinese adults. Br J Nutr. (2014) 112:1735–44. doi: 10.1017/s0007114514002657 25300318

[40] Chen YM Ho SC Lam SS Chan SS. Validity of body mass index and waist circumference in the classification of obesity as compared to percent body fat in Chinese middle-aged women. Int J Obes (2005). (2006) 30:918–25. doi: 10.1038/sj.ijo.0803220 16432548

[41] Dong D Lou P Wang J Zhang P Sun J Chang G. Interaction of sleep quality and anxiety on quality of life in individuals with type 2 diabetes mellitus. Health Qual Life outcomes. (2020) 18:150. doi: 10.1186/s12955-020-01406-z 32448338 PMC7247196

[42] Zhu S Zhao A Lan H Li P Mao S Szeto IM. Nausea and vomiting during early pregnancy among chinese women and its association with nutritional intakes. Nutrients. (2023) 15 933. doi: 10.3390/nu15040933 36839295 PMC9962185

[43] Mu L Yu F Xia J Lang X Haque A Wu HE. Association between high bmi and high homocysteine levels in chinese patients with bipolar disorder. J Affect Disord. (2021) 295:284–90. doi: 10.1016/j.jad.2021.08.032 34482060

[44] Lipsitz O McIntyre RS Rodrigues NB Lee Y Gill H Subramaniapillai M. Does body mass index predict response to intravenous ketamine treatment in adults with major depressive and bipolar disorder? results from the canadian rapid treatment center of excellence. CNS spectrums. (2022) 27:322–30. doi: 10.1017/S1092852920002102 33267928

[45] MaChado-Vieira R Gold PW Luckenbaugh DA Ballard ED Richards EM Henter ID. The role of adipokines in the rapid antidepressant effects of ketamine. Mol Psychiatry. (2017) 22:127–33. doi: 10.1038/mp.2016.36 PMC5112162 27046644

[46] Niciu MJ Luckenbaugh DA Ionescu DF Guevara S MaChado-Vieira R Richards EM. Clinical predictors of ketamine response in treatment-resistant major depression. J Clin Psychiatry. (2014) 75:e417–23. doi: 10.4088/JCP.13m08698 PMC4310499 24922494

[47] Dale RM Bryant KA Thompson NR. Metabolic syndrome rather than body mass index is associated with treatment response to ketamine infusions. J Clin psychopharmacol. (2020) 40:75–9. doi: 10.1097/JCP.0000000000001149 31834094

[48] Mannan M Mamun A Doi S Clavarino A. Prospective associations between depression and obesity for adolescent males and females- a systematic review and meta-analysis of longitudinal studies. PloS One. (2016) 11:e0157240. doi: 10.1371/journal.pone.0157240 27285386 PMC4902254

[49] Nigatu YT Reijneveld SA de Jonge P van Rossum E Bultmann U. The combined effects of obesity, abdominal obesity and major depression/anxiety on health-related quality of life: the lifeLines cohort study. PloS One. (2016) 11:e0148871. doi: 10.1371/journal.pone.0148871 26866920 PMC4750966

[50] Lu XY Kim CS Frazer A Zhang W. Leptin: a potential novel antidepressant. Proc Natl Acad Sci United States America. (2006) 103:1593–8. doi: 10.1073/pnas.0508901103 PMC1360555 16423896

[51] Milaneschi Y Lamers F Bot M Drent ML Penninx BW. Leptin dysregulation is specifically associated with major depression with atypical features: evidence for a mechanism connecting obesity and depression. Biol Psychiatry. (2017) 81:807–14. doi: 10.1016/j.biopsych.2015.10.023 26742925

[52] Rashidian H Rosenblat JD McIntyre RS Mansur RB. Leptin, obesity, and response to ketamine. Prog Neuropsychopharmacol Biol Psychiatry. (2020) 98:109773. doi: 10.1016/j.pnpbp.2019.109773 31672525

[53] Chen M-H Li C-T Lin W-C Hong C-J Tu P-C Bai Y-M. Rapid inflammation modulation and antidepressant efficacy of a low-dose ketamine infusion in treatment-resistant depression: a randomized, double-blind control study. Psychiatry Res. (2018) 269:207–11. doi: 10.1016/j.psychres.2018.08.078 30153598

[54] Zhou Y Wang C Lan X Li H Chao Z Ning Y. Plasma inflammatory cytokines and treatment-resistant depression with comorbid pain: improvement by ketamine. J neuroinflamm. (2021) 18:200. doi: 10.1186/s12974-021-02245-5 PMC8444441 34526064

[55] Park M Newman LE Gold PW Luckenbaugh DA Yuan P MaChado-Vieira R. Change in cytokine levels is not associated with rapid antidepressant response to ketamine in treatment-resistant depression. J Psychiatr Res. (2017) 84:113–8. doi: 10.1016/j.jpsychires.2016.09.025 PMC5125870 27718369

[56] Kruse JL Vasavada MM Olmstead R Hellemann G Wade B Breen EC. Depression treatment response to ketamine: sex-specific role of interleukin-8, but not other inflammatory markers. Trans Psychiatry. (2021) 11:167. doi: 10.1038/s41398-021-01268-z PMC7960960 33723220

